# High maternal pre-pregnancy BMI is associated with increased offspring peer-relationship problems at 5 years

**DOI:** 10.3389/frcha.2022.971743

**Published:** 2022-10-28

**Authors:** Courtney Dow, Elsa Lorthe, Cédric Galera, Muriel Tafflet, Laetitia Marchand-Martin, Pierre-Yves Ancel, Marie-Aline Charles, Barbara Heude

**Affiliations:** ^1^Centre for Research in Epidemiology and StatisticS (CRESS), Inserm, INRAE, Université Paris Cité, Paris, France; ^2^Unit of Population Epidemiology, Department of Primary Care Medicine, Geneva University Hospitals, Geneva, Switzerland; ^3^UMR 1219, Inserm, Univ. Bordeaux, Bordeaux Population Health Center, Bordeaux, France; ^4^Centre Hospitalier Perrens, Bordeaux, France; ^5^Unit on Children's Psychosocial Maladjustment, Université de Montreal, Montreal, QC, Canada

**Keywords:** maternal obesity, behavioral problems, pre-pregnancy, peer problems, lifecourse

## Abstract

**Background:**

Peer relationships are an important aspect of child development that are often overlooked. Maternal pre-pregnancy body mass index (BMI) may influence peer relationships through intrauterine mechanisms affecting fetal neurodevelopment or through postnatal mechanisms including social discrimination of the obese mother/child. This study aimed to determine the relationship between maternal pre-pregnancy BMI and child peer-relationship problems around 5 years old, including preterm and term-born children.

**Methods and findings:**

Maternal BMI and offspring peer-relationship problems were assessed in participants of three French birth cohorts: EDEN (*n* = 1,184 children born at term), ELFE (*n* = 10,889 children born ≥33 weeks of gestation) and EPIPAGE-2 (*n* = 2,646 children born 23–34 weeks of gestation). Reported or measured pre-pregnancy weight (kg) and height (m) were collected from mothers and used to calculate BMI (kg/m^2^). Offspring peer-relationship problems were assessed using the Strengths and Difficulties Questionnaire at 5.5 years. Logistic regression was used to estimate odds ratios (OR) of a high peer-relationship problem score (≥3) in EDEN and ELFE, and generalized estimated equations were used in EPIPAGE-2 to account for the large number of multiple births. Paternal BMI was used as a negative control in sensitivity analyses. Maternal pre-pregnancy obesity was associated with increased odds of a high peer-relationship problem score in all three cohorts, independent of confounding factors [adjusted OR 2.27 (1.32, 3.88); 1.52 (1.29, 1.78); 1.44 (1.04, 1.99); for EDEN, ELFE and EPIPAGE-2, respectively]. Additional analysis based on negative controls (i.e., adjusting for paternal BMI) showed the same pattern of associations.

**Conclusion:**

High maternal pre-pregnancy BMI is associated with greater likelihood of a high peer-relationship trouble score in offspring around 5 years of age in both children born preterm and at term.

## Introduction

The perinatal period is an extremely important timeframe for neurodevelopment, sensitive to both environmental and biological stressors. Such stressors are believed to impair normal growth and development and create the basis for the Developmental Origins of Health and Disease (DOHaD), an approach in public health research focused on the role of the pre- and perinatal environment in determining the development of diseases in adulthood [1]. Maternal pre-pregnancy obesity is a potential stressor on fetal development that has been exponentially increasing in prevalence worldwide [2]. The exact mechanisms through which maternal obesity may cause insults to the developing fetus remain to be elucidated. However, metabolic changes, inflammation, variations in the steroid or hormonal environment and induced epigenetic changes (that may occur as a consequence of the previous factors) have been identified as likely culprits [3]. In particular, large epidemiological studies have linked maternal pre-pregnancy obesity to a host of neurodevelopmental issues for the offspring including autism spectrum disorder, schizophrenia, cognitive impairment, attention deficit hyperactivity disorder (ADHD), depression and anxiety [3]. Nevertheless, whether maternal obesity affects the development of peer-relationship problems in childhood has not been studied in detail, despite the significance of peer relationships in healthy development.

Peer relationships are an important aspect of child development that often seems to be overlooked. Yet, they represent an “absolute necessity for healthy cognitive and social development and socialization” [4]. Studies show that early peer relationships affect early adulthood social adjustment. Correlations have been observed between early relationships and externalizing symptoms such as aggressive, suicidal, and illegal behavior [5–7] and internalizing problems such as anxiety and depression [8]. Children with good peer relationships also tend to accomplish more academically, have greater emotional wellbeing, and value prosocial behavior more strongly than children with troublesome peer relationships [5, 9].

Though several studies have examined the role of maternal pre-pregnancy obesity on child behavior in general, many do not discriminate between domains. Yet, in the few that have examined different aspects of behavior separately, the associations observed are usually far from uniform across domains [10–12]. With regards to peer-relationship problems, some prospective cohort studies have noted a positive association between maternal pre-pregnancy obesity and peer problems in childhood with up to twofold increased odds [10, 13], while others have observed no association [12, 14, 15] and in one, the association varied depending on the reporter (parents or teacher) [11]. All of these studies were quite limited in the confounding factors they accounted for, some of which have been quite consistently linked to child neurodevelopment, such as the maternal diet and maternal alcohol intake [16, 17]. Other factors are likely important candidates for confounding, such as maternal physical activity and maternal mental health [18, 19] and were also not taken into account. In addition, none of these studies were able to evaluate the same children across time, yet the severity of symptoms may vary with time and depend strongly on the types of environments children are exposed to at different ages. Finally, to our knowledge, there are no studies that have examined the impact of maternal pre-pregnancy obesity on the development of peer problems in preterm offspring, who represent a developmentally unique group already at increased risk of neurodevelopmental deficits, including behavioral problems, compared to children born at term [20, 21].

Thus, the objective of our study was to determine whether maternal pre-pregnancy body mass index (BMI) was associated with peer-relationship problems in children between the ages of 3–8 years in three French birth cohorts, one of which is a large cohort of infants born moderately to extremely preterm (<34 weeks gestation) while simultaneously adjusting for important potential confounders of the relationship such as maternal lifestyle behaviors (diet, exercise, alcohol and tobacco consumption) and maternal mental state during pregnancy.

## Materials and methods

### Study populations

#### EDEN

EDEN is a mother-child cohort initiated in the French cities of Nancy and Poitiers in 2003 [22]. Its goal was to study both pre- and post-natal determinants of child health and development. A total of 2002 expecting mothers (<24 weeks of gestation) agreed to participate in the study and were recruited from two maternity clinics if they had singleton pregnancies, no known diabetes before pregnancy, were French literate and had no plans to move out of the region in the following 3 years. During pregnancy, 95 of these mothers withdrew from the study (Supplementary Figure 1a). Data collection was *via* questionnaires, clinical examinations and cognitive assessments from pregnancy to 12 years of age and is still ongoing. At study enrollment, written informed consent was obtained from parents.

#### ELFE

ELFE (French Longitudinal Study since Childhood) is a nationwide mother-child cohort that was initiated in 2011 to study the determinants of child health, development and socialization from birth and throughout the lifecourse [23]. Maternities throughout metropolitan France were randomly selected and 320 (92%) agreed to participate. Recruitment took place over 25 days divided into four periods during the year. Mothers were approached after delivery if they had: birth(s) ≥33 weeks of gestation, were at least 18 years old, and had no plan to leave metropolitan France in the following 3 years. Consent was obtained from parents or the mother alone (with the father informed of his right to oppose) at enrollment and all documents were available in French, English, Arabic and Turkish.

A total of 18,040 mothers agreed to participate in ELFE and gave birth to 18,329 babies (Supplementary Figure 1b). Since enrollment, 59 women asked to withdraw from the study, giving rise to an initial study population of 18,270 children. Data collection was *via* phone interview, internet and paper questionnaires with one or both parents, home visits and physician-filled questionnaires. Biological samples were also collected, school medical examination records were obtained and nursery schoolteachers completed questionnaires.

#### EPIPAGE-2

EPIPAGE-2 is a population-based French cohort of infants born preterm designed to study the determinants of preterm birth, short and long-term outcomes and the impact of changes in provided care and practices on preterm infants [24]. Participants were recruited from March to December 2011 in all maternities throughout 25 of 26 regions of France. Live born, stillborn, and terminations of pregnancy between 22 and 34 weeks of gestation were eligible. Participants were classified into one of three gestational age groups: extremely preterm (22–26 weeks of gestation), very preterm (27–31 weeks), or moderately preterm (32–34 weeks) and longer recruitment periods were undertaken for smaller gestational age groups. A total of 7,804 were enrolled in EPIPAGE-2, of these, 4,467 infants were discharged from neonatal care alive and 177 withdrew (family refused or child died), resulting in 4,290 infants eligible for follow-up (Supplementary Figure 1c).

Data were collected at birth and during the neonatal period by questionnaires completed by the obstetrical and neonatal teams or extracted from medical records. Information in the follow-up period was collected through questionnaires completed by parents, physicians, and/or health assessments at regional exam centers.

### Ethics

Ethics approval for data collection in ELFE and EPIPAGE-2 was obtained from the French National Commission on Informatics and Liberty (CNIL), the Advisory Committee on Information Processing in Maternal Research in the Field of Health (CCTIRS) and the Committee for the Protection of People (CPP). The EDEN cohort was approved by the Bicêtre Hospital ethics committee and the CNIL.

### Exposure assessment

In EDEN, maternal height was measured by midwives and pre-pregnancy weight was self-reported between 24 and 26 weeks of gestation. In ELFE, information on pre-pregnancy weight and height were collected in face-to-face interviews or self-administered questionnaires at the time of delivery. In EPIPAGE-2, information about pre-pregnancy weight and height were extracted from medical records. Pre-pregnancy BMI was calculated and classified as: underweight (<18.5kg/m^2^), normal (18.5–24.9 kg/m^2^), overweight (25.0–29.9 kg/m^2^) and obese (≥30 kg/m^2^).

### Outcome assessment

The Strengths and Difficulties Questionnaires (SDQ) is a validated tool used to assess behavioral difficulties in children [25]. It includes five domains: peer-relationship troubles, hyperactivity-inattention, emotional symptoms, conduct problems and prosocial behavior. Each domain is comprised of 5 items, the following are the items included in the peer-relationship domain: (1) rather solitary, tends to play alone; (2) has at least one good friend; (3) generally liked by other children; (4) picked on or bullied by other children; (5) gets on better with adults than other children. The responses vary on a 3-point Likert scale from “Not true (0 points)” to “A little true (1 points) to “Very true (2 points).” This results in a score ranging from 0 to 10 for each domain.

The SDQ was administered to parents when children were aged 3, 5.5 and 8 in the EDEN cohort, and 5.5 years in ELFE and EPIPAGE-2. In ELFE, SDQ data were collected for 11,247 children and after excluding the small number of twins, the study sample was comprised of 10,889 children. In EPIPAGE-2, SDQ data were collected for 2,646 children. The mean scores were: 1.2, 1.2 and 1.5 for EDEN, ELFE and EPIPAGE-2, respectively. A score ≥90th percentile in EDEN and ELFE was considered “high-risk” for all three cohorts, which corresponded to a score ≥3. This threshold corresponded to the 88th percentile in EPIPAGE-2 and is consistent with the classic SDQ cut-off, which considers the extreme 10% of the population as the “high-risk” group [25].

### Covariates

Covariates collected from parents in all cohorts included parental: age, level of education, country of birth, employment status, household income or socioeconomic status, cohabitation and region of residence. Variables related to the pregnancy were: folic acid intake before conception, parity, gestational weight gain (GWG; EDEN/ELFE), gestational diabetes (GDM), pre-existing diabetes (ELFE), hypertension in pregnancy, pre-eclampsia, psychiatric problem before or during pregnancy (EDEN/ELFE) or anxiety score during pregnancy (EPIPAGE-2), maternity center, cause of prematurity (EPIPAGE-2), type of delivery, and duration of breastfeeding. Maternal lifestyle covariates encompassed: smoking in pregnancy, alcohol intake in pregnancy (EDEN/ELFE), dietary profile score during pregnancy (EDEN) [26]/score of adherence to the National Nutrition guidelines for pregnant women (ELFE) [27] and physical activity score [EDEN: *ad-hoc* from principal component analysis; ELFE: score derived from the Pregnancy Physical Activity Questionnaire = metabolic equivalents (METS)_activity_ × time (hours) × 7 days] [28]. Lastly, variables related to the child were: gestational age at birth, sex, birth weight, childcare from birth to 5 years, sleep duration at 2 years (EDEN/ELFE), night waking at 2 years (EDEN/EPIPAGE-2), difficulty falling asleep (ELFE only, at 2 years), duration of weekly screentime at 2 years (ELFE) and the HOME (Home Observation Measurement of the Environment) questionnaire (5.5 years) which measures the level of stimulation in the child's home environment (EDEN/EPIPAGE-2) [29].

### Statistical analyses

#### Missing data and weighting

Missing data were imputed using multiple imputation with chained equations, generating 60 datasets in EDEN, 45 in ELFE and 65 in EPIPAGE-2. The number of imputed datasets was chosen based off the fraction of missing information in the parameters included in the procedure. The variables included in the multiple imputation were all those listed previously. To impute categorical variables, the discriminant function method was employed, and predictive mean matching was used to impute quantitative variables.

We previously observed differences in attrition by baseline characteristics in EDEN [30]. Inverse probability weighting was used to correct this bias due to loss to follow-up. Logistic regression (EDEN and ELFE) or generalized estimating equations (GEE; to take into account the large proportion of multiple births; EPIPAGE-2) were fit for baseline data associated with not having available data for the SDQ (*p* < 0.20) to calculate stabilized weights. The best models were chosen based on parsimony and by comparing Akaike information criterion (AIC) between nested models.

#### Data analysis

Descriptive analyses were performed on all populations to describe the overall distribution of characteristics. In EPIPAGE-2, the descriptive statistics were weighted to account for the recruitment scheme. Characteristics of participants with low and high (≥3) peer-relationship trouble scores were compared using ANOVA or chi^2^ test, as appropriate.

A thorough review of the literature and the construction of a DAG identified potential confounding factors. Logistic regression was then used to analyze the association between maternal pre-pregnancy BMI and either a high score on the peer-relationship trouble domain (EDEN, ELFE, EPIPAGE-2) or a high peer-relationship trouble score trajectory (EDEN) for all children with follow-up. Models were adjusted for all potential confounding factors identified in the literature review and confounders were retained if they contributed to lowering the AIC, if they modified the beta coefficients (>10%) or if they were considered essential to adjust for (i.e., sex). All statistical analyses were completed in SAS 9.4 (SAS Institute Inc, Cary, NC).

### Sensitivity analyses

First, linear regression was conducted in all cohorts between maternal pre-pregnancy BMI and the continuous score on the SDQ peer-relationship problem domain. Next, complete case analyses were conducted using only mother-child pairs with no missing data for the exposure, outcome or covariates. Third, we tested for interactions with sex, gestational age, singleton pregnancy (ELFE and EPIPAGE-2) and cause of prematurity (EPIPAGE-2).

Fourth, paternal BMI can provide valuable information as a negative control. Using paternal BMI could provide support for a direct, causal association of maternal BMI if paternal BMI does not have a similar magnitude as maternal BMI with child peer-relationship problems [31]. We both adjusted for paternal BMI in the same model with maternal pre-pregnancy BMI, as well as ran analyses with paternal BMI separately, considered as the main exposure. Due to the high amount of missing data for paternal BMI in ELFE (31.0%) and EPIPAGE-2 (64.9%) we also ran complete-case analyses using only observations for which paternal BMI was not imputed.

Fifth, as we had SDQ data available at three time points in EDEN [3 (*n* = 1,307), 5.5 (*n* = 1,184), and 8 years (*n* = 875)], we conducted group-based trajectory modeling, which allowed the identification of specific clusters of children with similar developmental patterns over the time period. These clusters were not based on a set of pre-defined characteristics, but rather the scores of the SDQ [32]. Each child with at least one SDQ assessment was included, resulting in a study population of 1,428 children. The procedure used to derive the trajectories has been detailed previously [33]. Briefly, trajectories were derived using semi-parametric mixed models with censored-normal distributions. To identify the best models, the Bayesian Information Criterion was used to single out the ideal models with respect to the number of groups and polynomial order. The quality of the model was evaluated by maximizing the posterior probability of group membership according to recommendations (≥0.7) [32]. Three groups of peer-relationship problems were derived: low (17.7%), medium (75.5%), and high (6.8%), which were subsequently classified into high vs. other. We also conducted individual analyses in children at 3 and 8 years.

## Results

### Descriptive

The descriptive characteristics of the EDEN population are shown in Table 1A. Children with higher peer-relationship trouble scores were more likely to be the first born, to have mothers with psychological problems during pregnancy and to have a smaller gestational age. The descriptive characteristics of the ELFE and EPIPAGE-2 populations are displayed in Tables 1B,C. In both cohorts, children with higher peer-relationship problem scores at 5 years had slightly younger mothers, who were more likely foreign-born, with lower socioeconomic status, and primiparous. In ELFE, children with more peer-relationship problems were more likely born to underweight or overweight/obese mothers, who were more likely to have GDM, hypertension, psychological problems, smoke during pregnancy and have worse diet quality. The children were also more likely to have their parents care for them than professionals and to have a higher weekly duration of screentime and more sleep difficulties. In EPIPAGE-2, higher scores were observed more often in children with lower birthweight, who were born more premature, in mothers without normal BMI, in mothers without hypertension, and mothers with higher anxiety scores during pregnancy. The children were more often cared for by parents, had more frequent night waking at 2 years, and had less stimulating home environments.

**Table 1A:** Descriptive characteristics of the EDEN study population overall and by SDQ peer-relationship score at 5.5 years (*n* = 1,184).

**Variable**	**Study population *N* = 1,184**	**SDQ peer-relationship problem score** ***N*** **(%) or mean [STD]**		***p*** **-value^a^**
		**“Low-risk”**	**“High risk”**	
		***n*** **= 1,014**	***n*** **= 170**	
*Socioeconomic factors*				
Center (*n* = 1,184)				
Poitiers	629 (53.1)	540 (53.3)	89 (52.4)	0.83
Nancy	555 (46.9)	474 (46.7)	81 (47.6)	
Mother's age at delivery (years) (*n* = 1,184)	30.1 [4.7]	30.2 [4.6]	29.9 [5.1]	0.58
Maternal education (birth) (*n* = 1,180)				
High	715 (60.6)	632 (62.5)	83 (49.1)	< 0.01
Medium	414 (35.1)	339 (33.5)	75 (44.4)	
Low	51 (4.3)	40 (4.0)	11 (6.5)	
*Parental lifestyle/health characteristics*				
Parity (including stillbirths) (*n* = 1,182)				
0	549 (46.4)	458 (45.3)	91 (53.5)	0.08
1	415 (35.1)	370 (36.6)	45 (26.5)	
2	162 (13.7)	136 (13.4)	26 (15.3)	
≥3	56 (4.7)	48 (4.7)	8 (4.7)	
Maternal BMI category (*n* = 1,164)				
Underweight (<18.5 kg/m^2^)	91 (7.8)	77 (7.7)	14 (8.3)	<0.01
Normal (18.5–24.9 kg/m^2^)	763 (65.5)	666 (66.9)	97 (57.7)	
Overweight (25–29.9kg/m^2^)	207 (17.8)	176 (17.7)	31 (18.5)	
Obese (≥30 kg/m^2^)	103 (8.8)	77 (7.7)	26 (15.5)	
Paternal BMI category (*n* = 1,109)				
Underweight (<18.5 kg/m^2^)	9 (0.8)	7 (0.7)	2 (1.3)	0.43
Normal (18.5–24.9 kg/m^2^)	574 (51.8)	491 (51.7)	83 (52.2)	
Overweight (25–29.9 kg/m^2^)	425 (38.3)	370 (38.9)	55 (34.6)	
Obese (≥30 kg/m^2^)	101 (9.1)	82 (8.6)	19 (11.9)	
Smoking during pregnancy (*n* = 1,179)				
No	926 (78.5)	799 (79.0)	127 (76.0)	0.40
Yes	253 (21.5)	213 (21.0)	40 (24.0)	
Alcohol intake during pregnancy (*n* = 1,184)				
No	565 (47.7)	480 (47.3)	85 (50.0)	0.52
Yes	619 (52.3)	534 (52.7)	85 (50.0)	
Exercise during pregnancy (score) (*n* = 1,142)	0.0 [1.0]	0.1 [1.0]	−0.1 [1.0]	<0.01
Western diet during pregnancy (score) (*n* = 1,028)	−0.1 [0.9]	−0.1 [0.9]	0.0 [1.0]	0.05
Gestational weight gain (kg) (*n* = 1,161)	13.1 [4.8]	13.2 [4.6]	13.1 [5.5]	0.8
Any psychiatric disorder (during pregnancy) (*n* = 1,184)				
No	1,112 (93.9)	954 (94.1)	158 (92.9)	0.56
Yes	72 (6.1)	60 (5.9)	12 (7.1)	
*Child characteristics*				
Gestational age (*n* = 1,184)				
<37 weeks (preterm)	68 (5.7)	54 (5.3)	14 (8.2)	0.26
37–38 weeks (early term)	215 (18.2)	182 (18.0)	33 (19.4)	
≥39 weeks (term)	901 (76.1)	778 (76.7)	123 (72.3)	
Birth weight (g) (*n* = 1,184)	3,292 [515.7]	3,289 [501.6]	3,315 [594.1]	0.54
Sex of the child (*n* = 1,184)				
Male	626 (52.9)	532 (52.5)	94 (55.3)	0.49
Female	558 (47.1)	482 (47.5)	76 (44.7)	
Childcare (2 years) (*n* = 1,165)				
Parents	293 (25.2)	244 (24.5)	49 (29.2)	<0.01
Relatives	151 (13.0)	118 (11.8)	33 (19.6)	
Professional/childcare center	226 (19.4)	207 (20.8)	19 (11.3)	
Maternal assistant	495 (42.5)	428 (42.9)	67 (39.9)	
Age at 5 years follow-up (months) (*n* = 1,184)	67.1 [1.9]	67.1 [1.9]	67.2 [2.0]	0.45

^a^Difference among BMI categories, by ANOVA or by chi^2^ test.

**Table 1B:** Descriptive characteristics of the overall population and by SDQ peer-relationship trouble score in ELFE (*n* = 10,889).

**Variable**	**Study population *N* = 10,889**	**SDQ peer-relationship problem score** ***N*** **(%) or mean [STD]**		***p*** **-value^a^**
		**“Low-risk” *n* = 9,309**	**“High risk” *n* = 1,580**	
*Sociodemographics*				
Maternal age at child's birth (*n* = 10,849)	30.9 [4.7]	31.0 [4.6]	30.6 [5.1]	<0.01
Maternal education (birth) (*n* = 10,887)				
High	7,374 (67.7)	6,497 (69.8)	877 (55.5)	<0.001
Medium	3,036 (27.9)	2,470 (26.5)	566 (35.8)	
Low	477 (4.4)	340 (3.7)	137 (8.7)	
Monthly revenue (*n* = 10,399)				
1st quartile (lowest)	1,719 (16.5)	1,529 (17.1)	190 (12.9)	<0.001
2nd quartile	2,534 (24.4)	2,224 (24.9)	310 (21.0)	
3rd quartile	3,575 (34.4)	3,114 (34.9)	461 (31.3)	
4th quartile (highest)	2,571 (24.7)	2,058 (23.1)	513 (34.8)	
Parity (*n* = 10,749)				
0	4,963 (46.2)	4,146 (45.1)	817 (52.4)	<0.001
1	3,919 (36.5)	3,449 (37.5)	470 (30.2)	
2	1,400 (13.0)	1,212 (13.2)	188 (12.1)	
≥3	467 (4.3)	384 (4.2)	83 (5.3)	
*Parental health/lifestyle*				
Maternal BMI category (*n* = 10,743)				
Underweight (<18.5 kg/m^2^)	755 (7.0)	638 (6.9)	117 (7.5)	<0.001
Normal (18.5–24.9 kg/m^2^)	7,244 (67.4)	6,304 (68.6)	940 (60.4)	
Overweight (25–29.9 kg/m^2^)	1,774 (16.5)	1,481 (16.1)	293 (18.8)	
Obese (≥30 kg/m^2^)	970 (9.0)	763 (8.3)	207 (13.3)	
Paternal BMI category (*n* = 9,379)				
Underweight (<18.5 kg/m^2^)	72 (0.8)	61 (0.8)	11 (0.9)	0.10
Normal (18.5–24.9 kg/m^2^)	5,221 (55.7)	4,540 (56.1)	681 (52.9)	
Overweight (25–29.9 kg/m^2^)	3,371 (35.9)	2,900 (35.8)	471 (36.6)	
Obese (≥30 kg/m^2^)	715 (7.6)	590 (7.3)	125 (9.7)	
Gestational weight gain (kg) (*n* = 10,676)	13.2 [5.2]	13.2 [5.1]	13.0 [5.7]	0.11
Any psychiatric disorder (during pregnancy) (*n* = 10,791)				
No	9,479 (87.8)	8,168 (88.5)	1,311 (83.8)	<0.001
Yes	1,312 (12.2)	1,058 (11.5)	254 (16.2)	
Smoking in pregnancy (*n* = 10,786)				
No	9,006 (83.5)	7,752 (84.0)	1,254 (80.3)	<0.001
Yes	1,780 (16.5)	1,473 (16.0)	307 (19.7)	
Alcohol intake in pregnancy (*n* = 10,147)				
None	6,097 (60.1)	5,154 (59.3)	943 (64.5)	<0.001
Light (<3 units)	3,966 (39.1)	3,465 (39.9)	501 (34.3)	
Moderate/heavy (≥3 units)	84 (0.8)	67 (0.8)	17 (1.2)	
Pregnancy diet quality score (*n* = 9,791)	7.7 (0.8)	7.7 (0.8)	7.6 (0.8)	<0.001
Physical activity score in 3rd trimester (*n* = 9,848)	174.5 (84.7)	173.6 (81.9)	179.6 (99.9)	0.01
*Child characteristics*				
Gestational age (*n* = 10,580)				
<37 weeks (preterm)	771 (7.1)	664 (7.1)	107 (6.8)	0.36
37–38 weeks (early term)	2,082 (19.1)	1,798 (19.3)	284 (18.0)	
*(Continued)*				
≥39 weeks (term)	8,036 (73.8)	6,847 (73.6)	1,189 (75.3)	
Birth weight (g) (*n* = 10,526)	3,339 [478.7]	3,339 [480.7]	3,339 [466.9]	0.99
Sex of child (*n* = 10,639)				
Male	5,519 (51.9)	4,733 (52.0)	786 (50.9)	0.39
Female	5,120 (48.1)	4,361 (48.0)	759 (49.1)	
Childcare at 2 years (*n* = 10,399)				
Parents	2,628 (25.3)	2,141 (24.0)	487 (32.7)	<0.001
Relatives	779 (7.5)	669 (7.5)	110 (7.4)	
Professional	4,592 (44.2)	4,004 (44.9)	588 (39.5)	
Childcare center	2,400 (23.1)	2,096 (23.5)	304 (20.4)	
Child's age at 5 years follow-up (*n* = 10,889)	66.5 [1.8]	66.5 [1.8]	66.4 [1.8]	0.04

a Difference by peer-relationship trouble score, by ANOVA or by chi^2^ test.

**Table 1C:** Descriptive characteristics of the overall population and by SDQ peer-relationship trouble score in the EPIPAGE-2 study (*n* = 2,646) weighted by gestational age group.

**Variable**	**Study population *N* = 2,646**	**SDQ peer-relationship problem score** ***N*** **(%) or mean [STD]**		***p*** **-Value^a^**
		**“Low-risk” *n* = 2,056**	**“High risk” *n* = 590**	
*Sociodemographics*				
Maternal age at child's birth (*n* = 2,646)	30.6 [0.14]	30.7 [0.15]	30 [0.33]	<0.01
Maternal education (birth) (*n* = 2,569)				
High	1,351 (54.0)	1,102 (57.7)	249 (39.5)	<0.001
Medium	528 (20.4)	407 (20.0)	122 (22.4)	
Low	690 (25.6)	490 (22.3)	197 (38.1)	
Household SES (*n* = 2,541)				
Managerial	661 (27.3)	553 (29.4)	107 (18.7)	<0.001
Intermediate	632 (25.8)	504 (27.4)	128 (19.5)	
Administrator, director, civil servant, student	647 (25.2)	496 (24.1)	151 (29.5)	
Domestic or sales employee	309 (11.3)	218 (10.1)	90 (16.3)	
Laborer	249 (8.7)	176 (7.9)	73 (12.1)	
Without profession	43 (1.7)	26 (1.1)	17 (4.0)	
Parity (*n* = 2,622)				
0	1,502 (56.8)	1,159 (56.2)	341 (58.7)	0.72
1	632 (25.4)	503 (25.9)	130 (24.2)	
≥2	488 (17.8)	376 (17.9)	111 (17.1)	
*Parental health/lifestyle*				
Maternal BMI category (*n* = 2,465)				
Underweight (<18.5 kg/m^2^)	180 (6.7)	130 (6.4)	50 (8.0)	<0.01
Normal (18.5–24.9 kg/m^2^)	1,430 (58.8)	1,148 (60.6)	281 (51.7)	
Overweight (25–29.9 kg/m^2^)	488 (20.1)	384 (20.3)	105 (19.8)	
Obese (≥30 kg/m^2^)	367 (14.3)	257 (12.7)	108 (20.6)	
Paternal BMI category (*n* = 975)				
Underweight (<18.5 kg/m^2^)	5 (0.4)	2 (0.4)	3 (0.7)	0.18
Normal (18.5–24.9 kg/m^2^)	545 (56.7)	435 (58.6)	110 (49.6)	
Overweight (25–29.9 kg/m^2^)	323 (30.7)	237 (30.1)	86 (33.1)	
Obese (≥30 kg/m^2^)	102 (12.1)	73 (10.9)	28 (16.6)	
Maternal smoking in pregnancy (*n* = 2,556)				
No	2,080 (83.2)	1,621 (83.4)	457 (82.1)	0.58
Yes	476 (16.8)	362 (16.6)	114 (17.9)	
Anxiety score in pregnancy (STAI-T) (*n* = 1,781)				
Weak	1,409 (81.7)	1,135 (84.3)	275 (70.6)	<0.001
Moderate	222 (11.0)	162 (9.5)	59 (17.3)	
High	150 (7.3)	106 (6.2)	44 (12.1)	
*Pregnancy/delivery*				
Gestational age (*n* = 2,646)				
>32–34 weeks	658 (64.3)	536 (26.1)	123 (20.8)	<0.01
27–31 weeks	1,659 (31.2)	1,280 (62.3)	378 (64.1)	
23–26 weeks	329 (4.6)	240 (11.7)	89 (15.1)	
Birth weight (g) (*n* = 2,646)	1,712 [12.5]	1,724 [13.7]	1,666 [29.5]	0.02
Cause of prematurity (*n* = 2,415)				
Preterm labor	1,086 (48.4)	856 (48.6)	229 (47.1)	0.46
PPROM	592 (23.5)	464 (23.9)	127 (21.5)	
Vascular pathology, isolated placental abruption or isolated IUGR	737 (28.2)	561 (21.1)	176 (31.3)	
*Child characteristics*				
Sex of child (*n* = 2,646)				
Male	1,421 (55.5)	1,085 (54.9)	336 (58.2)	0.30
Female	1,225 (44.5)	971 (45.1)	254 (41.8)	
Childcare at 2years (*n* = 2,490)				
Parents	1,022 (38.9)	758 (37.2)	265 (46.3)	<0.001
Relatives/employee at home	173 (6.9)	135 (7.2)	38 (5.8)	
Professional/childcare center	423 (18.0)	336 (18.4)	86 (16.2)	
Maternal assistant	626 (27.9)	523 (29.8)	103 (20.3)	
Other	246 (8.2)	186 (7.4)	58 (11.4)	
Child's age at 5 years follow-up (*n* = 2,646)	67.3 [0.1]	67.3 [0.1]	67.4 [0.1]	0.39

a Difference by peer-relationship trouble score, by ANOVA or by chi^2^ test.

PPROM, preterm premature rupture of the membranes; STAI-T, State-Trait Anxiety Inventory; IUGR, intrauterine growth restriction.

### Maternal BMI and offspring peer-relationship problems

In unadjusted logistic regression, maternal pre-pregnancy obesity was associated with more than two-fold increased odds of a high peer-relationship problem score at 5.5 years in EDEN and two-fold increased odds of a high score at 5.5 years in ELFE (Table 2). The magnitude of the unadjusted relationship in infants born premature was relatively smaller [OR 1.62 (1.19, 2.20)]. In all three cohorts, adjustment for covariates slightly attenuated the magnitude of the association, however, the association remained statistically significant in the final models.

**Table 2:** Association between maternal pre-pregnancy body mass index and high peer-relationship problem scores at 5.5 years in the EDEN, ELFE, and EPIPAGE-2 cohorts.

**Variable**	**Unadjusted OR [95%CI]**	**Model 1^a^ [95%CI]**	**Model 2^b^ [95%CI]**	**Model 3^c^ [95%CI]**
*EDEN (n = 1,184)*
Obese (≥30 kg/m^2^)	2.30 [1.38, 3.82]	2.28 [1.35, 3.84]	2.25 [1.33, 3.81]	2.27 [1.32, 3.88]
Overweight (25–29.9 kg/m^2^)	1.13 [0.72, 1.77]	1.08 [0.68, 1.71]	1.09 [0.69, 1.73]	1.05 [0.66, 1.67]
Normal (18.5–24.9 kg/m^2^)	REF	REF	REF	REF
Underweight (<18.5 kg/m^2^)	1.25 [0.70, 2.21]	1.18 [0.65, 2.13]	1.10 [0.60, 2.00]	1.15 [0.63, 2.11]
*ELFE (n = 10,889)*
Obese (≥30 kg/m^2^)	1.93 [1.65, 2.25]	1.57 [1.34, 1.85]	1.53 [1.30, 1.80]	1.52 [1.29, 1.78]
Overweight (25–29.9 kg/m^2^)	1.36 [1.19, 1.56]	1.22 [1.07, 1.41]	1.21 [1.05, 1.39]	1.20 [1.05, 1.39]
Normal (18.5–24.9 kg/m^2^)	REF	REF	REF	REF
Underweight (<18.5 kg/m^2^)	1.40 [1.16, 1.69]	1.29 [1.06, 1.56]	1.30 [1.08, 1.58]	1.30 [1.08, 1.58]
*EPIPAGE-2 (n = 2,646)*
Obese (≥30 kg/m^2^)	1.62 [1.19, 2.20]	1.45 [1.06, 1.99]	1.44 [1.05, 1.98]	1.44 [1.04, 1.99]
Overweight (25–29.9 kg/m^2^)	1.07 [0.81, 1.42]	1.01 [0.75, 1.35]	1.00 [0.75, 1.33]	1.03 [0.77, 1.38]
Normal (18.5–24.9 kg/m^2^)	REF	REF	REF	REF
Underweight (<18.5 kg/m^2^)	1.67 [1.08, 2.57]	1.46 [0.95, 2.24]	1.48 [0.96, 2.28]	1.51 [0.98, 2.31]

a Adjusted for study center (EDEN), maternal education, household monthly income (ELFE), household socioeconomic status (EPIPAGE-2), parity, sex, psychological problems during pregnancy (EDEN+ELFE), maternal age at birth, singleton pregnancy (EPIPAGE-2).

b Additionally adjusted for maternal physical activity during pregnancy (EDEN + ELFE), maternal diet in pregnancy (EDEN + ELFE), maternal alcohol intake during pregnancy (EDEN +ELFE), maternal smoking during pregnancy, maternal anxiety during pregnancy (EPIPAGE-2).

c Additionally adjusted for gestational age, child age at evaluation, childcare at 2 years, cause of prematurity (EPIPAGE-2).

Maternal pre-pregnancy overweight was not associated with high offspring peer-relationship problem scores in EDEN or EPIPAGE-2 [aOR 1.06 (0.66, 1.69); aOR 1.03 (0.77, 1.38), respectively]. In ELFE, maternal overweight was associated with 20% increased odds of a high peer-relationship problem score at 5.5 years in adjusted analyses.

Maternal pre-pregnancy underweight was not associated with peer-relationship problem scores in EDEN [aOR 1.14 (0.61, 2.13)], but it was associated with 30% increased odds of a high peer-relationship problem score in ELFE and near the limit of statistical significant in EPIPAGE-2 [aOR 1.51 (0.98, 2.31)].

### Sensitivity analyses

Linear analyses in both EDEN and ELFE supported a positive association between maternal pre-pregnancy obesity and high offspring peer-relationship problem scores around 5 years of age (Supplementary Table 1). In EPIPAGE-2, a positive relationship between maternal obesity and increasing peer-problem scores was observed in univariate analyses but attenuated below the threshold of statistical significance after adjustment for confounders [*a*β 0.19 (−0.04, 0.41)]. Maternal overweight was not associated in linear analyses with increasing peer-relationship problem scores in any of the three cohorts. But, maternal underweight was associated with increasing peer-relationship problem scores in ELFE [*a*β 0.12 (0.02, 0.23)] and showed a higher, though non-statistically significant magnitude in EPIPAGE-2 [*a*β 0.24 (−0.06, 0.52)].

In complete-case analyses the associations observed changed little from those observed in the main results (Supplementary Table 2). None of the interactions tested were significant in EDEN or ELFE (*p* > 0.10). On the other hand, in EPIPAGE-2, we observed a significant interaction between maternal BMI and the cause of prematurity (*p* = 0.04; Supplementary Table 3). When the cause of prematurity was due to a vascular pathology, isolated placental abruption or isolated intrauterine growth restriction (IUGR) the odds of an increased peer problem score in offspring of both underweight and obese mothers was much higher [aOR 3.16 (1.22, 8.91); 2.57 (1.57, 4.21) for underweight and obese, respectively]. There was no association between maternal pre-pregnancy BMI and offspring peer-relationship problems in women with preterm labor [aOR 1.06 (0.58, 1.94); 1.37 (0.78, 2.41) for underweight and obese, respectively]. On the other hand, we observed an inverse, though non-statistically significant, association between pre-pregnancy obesity and offspring peer-relationship problems when the cause of prematurity was preterm premature rupture of the membranes [PPROM; aOR 0.50 (0.23, 1.06)].

**Figure 1:**
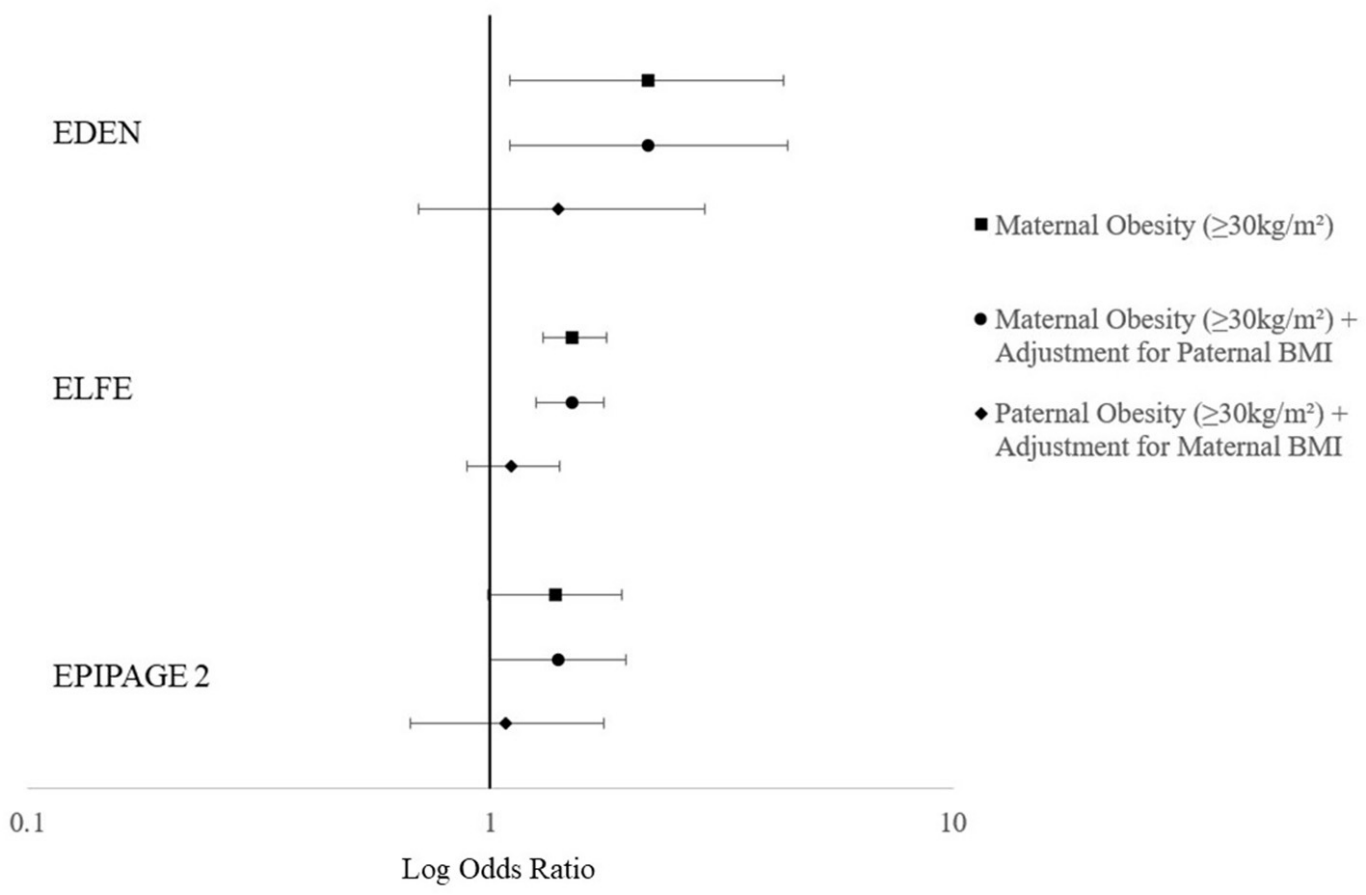
Adjusted^a^ odds ratios for a high peer-relationship score trajectory from 3 to 8 years (EDEN, *n* = 1,428), a high peer-relationship score at 5.5 years (ELFE, *n* = 10,889) and a high peer-relationship score at 5 years in EPIPAGE-2 (*n* = 2,646). ^a^Adjusted for study center (EDEN), maternal education, household monthly income (ELFE), household socioeconomic status (EPIPAGE-2), parity, sex, psychological problems during pregnancy (EDEN + ELFE), maternal age at birth, singleton pregnancy (EPIPAGE-2), maternal physical activity during pregnancy (EDEN + ELFE), maternal diet in pregnancy (EDEN + ELFE), maternal alcohol intake during pregnancy (EDEN + ELFE), maternal smoking during pregnancy, maternal anxiety during pregnancy (EPIPAGE-2), gestational age, child age at evaluation, childcare at 2 years, cause of prematurity (EPIPAGE-2) and paternal BMI.

Adjustment for paternal BMI did not change estimates in any cohort (Figure 1). When paternal BMI was modeled separately without maternal BMI it was not associated with offspring peer-relationship problems [aOR 1.40 (0.69, 2.88); aOR 1.20 (0.96, 1.51); aOR 1.13 (0.70, 1.82); for paternal obesity in EDEN, ELFE, and EPIPAGE-2, respectively].

Similar relationships between maternal obesity and underweight with offspring peer-relationship problem score trajectories (3–8 years) were observed as those at 5.5 years in EDEN (Supplementary Table 4). The magnitude of the relationship with maternal overweight was higher but statistically non-significant with the 3–8 year peer-relationship problem score trajectories. In individual analyses at 3 years in EDEN, none of the maternal pre-pregnancy BMI classes were associated with high offspring peer-relationship problem scores. At 8 years, none of the estimates reached statistical significance but were similar in magnitude to those estimated in ELFE at 5.5 years.

## Discussion

Using data from three French birth cohort studies, including a large study of infants born premature, we found that children whose mothers were obese before pregnancy were much more likely to have adverse peer-relationship problems at 5 years of age than children whose mothers had a healthy pre-pregnancy BMI. The fact that we observed this relationship in three separate cohorts with children around the same age, using both clinically significant thresholds and continuous scores further strengthens our results. Our findings were also robust to adjustment for a variety of important confounding factors that previous studies did not take into account, such as maternal diet, physical activity, and alcohol intake. Paternal BMI was not associated with peer-relationship problem scores and did not change our estimates when it was included in the model, in favor of a direct intrauterine effect of maternal BMI on the offspring rather than residual confounding by genetics or the postnatal environment.

Our findings are consistent with previous studies that also observed an increased risk of social problems in children born to mothers with pre-pregnancy obesity [10, 11, 13]. However, not all of these studies are completely in accordance. Jo et al. [10] only observed this relationship among mothers in obesity class II/III (≥35 kg/m^2^) and the relationship showed no association with obese class I [OR = 1.01 (0.57, 1.78)]. On the other hand, Mikkelsen et al. [13] observed a significant association in both overweight and obese mothers. Menting et al. [11] only reported a significant association between teacher-rated peer-relationship problems and maternal pre-pregnancy obesity, but not mother-rated social problems. Finally, several other studies did not observe any relationship with maternal obesity and peer-relationship problems in offspring [12, 14, 15]. One reason for the variability in the results could be the age of evaluation, ages ranged from 4 to 8 years and a child at ages 3/4 vs. 5/6 or 7/8 years old are at very different developmental stages with regards to social and emotional development [34]. Indeed, we observed no association at 3 years in EDEN, despite observing a non-statistically significant association at 8 years of similar magnitude to that of ELFE and EPIPAGE-2 at 5 years. There may also be disparity in the results due to the wide range of countries across these studies, as the impact of maternal obesity may differ across ethnic groups [35].

In EDEN, we observed the strongest association between high maternal pre-pregnancy BMI and offspring peer problems at 5.5 years. Peer problems may not yet be as evident in children as young as 3 years old, as social development is still quite immature. To our knowledge, the youngest age that peer problems have been examined in association with maternal pre-pregnancy BMI is around 4 years old [12]. This study did not observe a relationship between mothers with severe pre-pregnancy obesity (≥40 kg/m^2^) and offspring peer-relationship problems, however, the cohort was relatively small. On the other hand, at 8 years, our results suggest a tendency toward increased peer-relationship problems that was not statistically significant. This could be due to a lack of statistical power in our 8-year population, but Robinson et al. [14] did not observe a relationship between obesity or severe obesity and child-relationship problems in children at 7–8 years. Conversely, Menting et al. [11] observed increased likelihood of teacher-reported peer-relationship problems in children between 5 and 7 years. In preterm children, socio-emotional or internalizing problems evaluated at 2 years old have been found to predict peer relationship problems at 5 years and psychiatric diagnoses at 11 years [21]. More studies are required to examine the longitudinal effects of maternal pre-pregnancy BMI on peer-relationship problems.

Only one other study that found a link between maternal obesity and offspring peer-relationship problems was able to use paternal BMI as a negative control. In this large Danish cohort, an association of similar magnitude was observed with both maternal and paternal BMI and peer-relationship problems [13]. We found that adjustment for paternal BMI did not change the association between maternal BMI and offspring peer-relationship problems nor was paternal BMI independently associated with peer-relationship troubles in our imputed and weighted models, in favor of a direct intrauterine effect of maternal BMI rather than residual confounding by genetics or the environment. Other studies have not observed a significant association between paternal BMI and offspring behavior [14, 36, 37] but one found a stronger, though non-significant, relationship with paternal than maternal BMI [38]. A recent Mendelian randomization study supports the hypothesis the link between maternal obesity and offspring hyperactivity-inattention symptoms may have both genetic and environmental origins [39], however the association with peer-relationship problems has not been investigated using genetic markers.

In ELFE, maternal underweight was associated with increased odds of high peer-relationship problem scores. There exists little literature on the role of maternal underweight in offspring peer problems as many studies do not analyze the role of maternal underweight at all [11, 12, 36, 37] or group it with normal BMI [14]. However, maternal underweight has been associated with twofold increased odds of teacher-rated difficulties in group situations at 5 years in one study [15], though not with peer-relationship problem scores at 6 years in another [10]. Results are also conflicting concerning other behavioral outcomes. In other studies, it has not been associated with problem behaviors in children 8–9 years old [35] but has been associated with externalizing problems in boys at 9–11 [40]. The results are equally contradictory concerning the association of maternal underweight with cognitive or psychomotor developmental outcomes. In some studies, there is no association between maternal underweight and cognitive or psychomotor development in offspring [38], but others find it associated with decreased cognitive scores and suggest there may be a U-shaped association between BMI and cognitive outcomes [41, 42]. Maternal underweight may be an indicator for inadequate prenatal micronutrient status and has been linked with fetal growth restriction, preterm birth, low birth weight, and increased risk of undernutrition in offspring, all of which are strong risk factors for adverse neurodevelopment [43, 44]. The role of maternal underweight represents an important avenue for future studies to investigate.

To our knowledge, this is the first study analyzing the relationship between maternal pre-pregnancy BMI and child peer-relationship problems in preterm infants. However, one previous study has analyzed the relationship between maternal pre-pregnancy BMI and offspring hyperactivity-inattention symptoms in children born preterm. In a population of 10-year-old children born extremely preterm (<28 weeks gestation), Van der Burg et al. [45] observed an increased risk in parent-rated, but not teacher-rated, hyperactivity-inattention symptoms for children whose mothers were overweight or obese before pregnancy. Children born preterm are a unique group already at increased risk of neurodevelopmental deficits compared to children born at term [20]. It is pertinent to determine whether high maternal pre-pregnancy BMI confers an additional, preventable risk to an already high-risk group. The risk of neurodevelopmental deficits appears to increase in a dose-response manner with decreasing gestational age [20]. However, with regards to peer-relationship problems, we did not find any interactions with gestational age in either EPIPAGE-2 or ELFE. This phenomenon could be explained by the fact that maternal obesity is also associated with increased neonatal mortality, which also rises with decreasing gestational age [46]. The competing risk of neonatal mortality may diminish the observed magnitude of association between maternal obesity and offspring behavioral deficits in preterm infants. As obese women are also at increased risk of preterm birth [47], the observed association maybe greater than what we have estimated.

We observed an interaction between the cause of prematurity and maternal pre-pregnancy BMI in EPIPAGE-2, suggesting that the positive association observed with the extremes of maternal BMI are driven by premature births caused by vascular pathologies, isolated placental abruption, or isolated IUGR. The association with premature labor was not statistically significant and conversely, PPROM showed a non-significant negative association. We are unsure how to explain this negative association, as infants born PPROM usually show similar risks of neonatal morbidity as those born due to spontaneous preterm labor and higher risks of acute antenatal complications and infections [48, 49].

It is believed that maternal obesity may directly affect the developing fetus through inflammation. Obese women have higher levels of proinflammatory factors than women of normal weight, and proinflammatory cytokines have been linked to oxidative stress in the placenta, placental inflammation and changes in fetal gene expression [3]. Inflammation has been found to disturb brain development and evidence suggests that multiple or sustained intermittent episodes of perinatal inflammation are more damaging to the brain than a single episode [50]. However, there are other mechanisms through which maternal obesity may influence behavioral development in offspring, including changes in the steroid or hormonal environment and behaviourally induced epigenetic modification [3, 51]. High paternal BMI may also contribute to increased risk of neurodevelopmental problems through epigenetic modification, though our results did not support this mechanism [52]. A few studies have attempted to determine the mechanisms of action. Both cytokine levels in cord blood and at 5 years old have been associated with behavioral problems, including peer-relationship issues, in children at 5 years in the EDEN cohort [53, 54]. In extremely preterm infants, levels of proinflammatory cytokines were higher in infants born to obese or overweight mothers in babies with induced preterm birth than infants delivered spontaneously [55]. This could indicate a contribution of maternal BMI to an inflammatory state, but possibly represents another competing risk situation [55]. Finally, one study observed an association between increasing cord blood leptin and decreased hyperactivity-inattention symptoms at 5 years, but no association with inflammatory cytokines such as TNF-α and IL-6 [56]. Further investigation into the roles of possible mediating factors is warranted, especially as the results concerning leptin are contrary to expected.

Other mechanisms of action between maternal obesity and child peer-relationship problems may not be as biological in nature. Social stigmatization of obese parents may play a role, and mothers may be subject to more stigma than fathers [57]. Social stigma may act by inducing psychological stress and consequently impacting fetal development in utero [58, 59] or maladaptive social networks and supports experienced by the parents may also be experienced by the offspring. The maternal social network has been significantly associated with offspring cognitive development at 2 years and thus may extend to child social development [60], possibly by providing more opportunities for playdates or social activities. In addition, as children of overweight mothers are more likely to be overweight themselves [61] they may be directly subject to increased social stigma by their peers [62]. Future studies are needed to quantify the role of child BMI as a potential mediator.

Our study has some limitations. Pre-pregnancy weight was self-reported in EDEN and ELFE and may have induced measurement error. However, weight is more likely to be under-reported than over-reported and would have attenuated our estimates rather than inflate them [63]. Ascertainment of peer-problems relied on parental reporting and teacher input may be valuable as they tend to observe children in much different social settings than parents. Indeed, poor/fair agreement between parent and teacher ratings have been reported for the SDQ [64]. However, the highest agreement between parents and teachers were observed for the peer-relationship problem and hyperactivity-inattention scales and the SDQ has proven a validated and reliable tool to distinguish behavioral problems [64, 65]. Finally, despite our adjustments we cannot rule out residual confounding.

Our study also has several strengths. We were able to evaluate an important aspect of behavioral development across three large birth cohorts, using the same tool, and around the same age, while adjusting our models for a wide range of important confounding factors not previously available to other studies. To our knowledge, this was the first study to evaluate the role of maternal BMI on social development in preterm infants and we were able to use paternal BMI as a negative control. Multiple imputation and inverse probability weighting also allowed us to increase efficiency, statistical power, and reduce bias due to attrition.

In conclusion, we observed that maternal pre-pregnancy obesity was associated with increased peer-problem scores at 5 years in three large birth cohorts, including a large cohort of infants born preterm. This association was not influenced by adjustment for maternal lifestyle or psychological problems. However, residual confounding by the postnatal environment or genetics may explain part of the association we observed. Given the current body of evidence, potential mediating factors and Mendelian randomization could support whether this association is causal in nature.

Efforts should be strengthened to ensure women of childbearing age have healthy weights before pregnancy, both with respect to reproductive counseling and with respect to more general healthy weight-based public health interventions. As randomized controlled trials have shown that behavioral and social skills of children can be ameliorated with intervention, healthcare providers should also be made aware of the potentially increased risk of pre-pregnancy obesity on child peer-relationship problems [21]. This could allow more timely identification of children at risk of behavioral problems and can provide more opportunities to improve their social skills during what may be the most modifiable period of their lives.

## Data availability statement

The datasets generated during and analyzed during the current study are not publicly available due to privacy laws set by the Commission nationale de l'informatique et des libertés (CNIL). Anonymized data may be made available upon reasonable request to any public or private research team and with permission of the EDEN, ELFE and EPIPAGE 2 scientific committees Requests to access the datasets should be directed at: Data requests concerning EDEN can be made through the website: http://eden.vjf.inserm.fr/en/page/25/submit-a-research-project. Data requests concerning ELFE can be made through the website: https://pandora-elfe.inserm.fr/public/index.php. Data requests concerning EPIPAGE 2 can be made using through the email: accesdonnees.epipage@inserm.fr by using information provided on the data access website: https://epipage2.inserm.fr/index.php/en/related-research/265-data-access-and-questionnaires.

## Ethics statement

The studies involving human participants were reviewed and approved by French National Commission on Informatics and Liberty (CNIL), and/or the Advisory Committee on Information Processing in Maternal Research in the Field of Health (CCTIRS), Committee for the Protection of People (CPP), or the Bicêtre Hospital Ethics Committee. Written informed consent to participate in this study was provided by the participants' legal guardian/next of kin.

## Author contributions

Conceptualization: CD, EL, CG, M-AC, and BH. Data curation: CD, MT, and LM-M. Formal analysis and writing original draft: CD. Funding acquisition: CD, P-YA, M-AC, and BH. Investigation and methodology: CD, EL, M-AC, and BH. Writing (reviewing and editing): CD, EL, CG, MT, LM-M, P-YA, M-AC, and BH. All authors contributed to the article and approved the submitted version.

## Funding

CD's salary was funded by a grant from the *Fondation pour la Recherche Médicale* (the French Foundation for Medical Research): SPF201909009122. The EDEN study acknowledges the following sources of funding: Foundation for Medical Research (FRM), National Agency for Research (ANR), National Institute for Research in Public Health (IRESP: TGIR cohorte santé 2008 program), French Ministry of Health (DGS), French Ministry of Research, Inserm Bone and Joint Diseases National Research (PRO-A) and Human Nutrition National Research Programs, Paris–Sud University, Nestle', French National Institute for Population Health Surveillance (InVS), French National Institute for Health Education (INPES), the European Union FP7 programmes (FP7/2007–2013, HELIX, ESCAPE, ENRIECO, Medall projects), Diabetes National Research Program [through a collaboration with the French Association of Diabetic Patients (AFD)], French Agency for Environmental Health Safety (now ANSES), Mutuelle Générale de l'Education Nationale (MGEN), French National Agency for Food Security, and the French-speaking association for the study of diabetes and metabolism (ALFEDIAM). Funders had no influence of any kind on analyses or result interpretation. The Elfe survey is a joint project between the French Institute for Demographic Studies (INED) and the National Institute of Health and Medical Research (INSERM), in partnership with the French blood transfusion service (Etablissement français du sang, EFS), Santé publique France, the National Institute for Statistics and Economic Studies (INSEE), the Direction générale de la santé (DGS, part of the Ministry of Health and Social Affairs), the Direction générale de la prévention des risques (DGPR, Ministry for the Environment), the Direction de la recherche, des études, de l'évaluation et des statistiques (DREES, Ministry of Health and Social Affairs), the Département des études, de la prospective et des statistiques (DEPS, Ministry of Culture), and the Caisse nationale des allocations familiales (CNAF), with the support of the Ministry of Higher Education and Research and the Institut national de la jeunesse et de l'éducation populaire (INJEP). Via the RECONAI platform, it receives a government grant managed by the National Research Agency under the Investissements d'avenir programme (ANR-11-EQPX-0038 and ANR-19-COHO-0001). EPIPAGE 2: The French National Institute of Public Health Research (IRESP TGIR 2009–01 programme)/Institute of Public Health and its partners [the French Health Ministry, the National Institute of Health and Medical Research (INSERM), the National Institute of Cancer, the National Solidarity Fund for Autonomy (CNSA)], the National Research Agency through the French programme of investments for the future (grants no. ANR-11-EQPX-0038 and ANR-10-COHO-0001) and the PremUp Foundation. Additional funding was obtained from the Fondation pour la Recherche Médicale (SPF 20160936356) and Fondation de France [00050329 and R18202KK (Grand Prix)].

## Conflict of interest

The authors declare that the research was conducted in the absence of any commercial or financial relationships that could be construed as a potential conflict of interest.

## Publisher's note

All claims expressed in this article are solely those of the authors and do not necessarily represent those of their affiliated organizations, or those of the publisher, the editors and the reviewers. Any product that may be evaluated in this article, or claim that may be made by its manufacturer, is not guaranteed or endorsed by the publisher.
