# Supplementary material for: High maternal pre-pregnancy BMI is associated with increased offspring peer-relationship problems at 5 years
Source: Front Child Adolesc Psychiatry. 2022 Oct 28;1:971743. doi: 10.3389/frcha.2022.971743 (PMC11731962; doi:10.3389/frcha.2022.971743)
Supplement: Supplementary file 1 [file Table_1.docx]

Supplementary Material

**Supplementary Table 1.** Linear association between maternal pre-pregnancy BMI classes and peer-relationship problem scores at 5.5 years in the EDEN, ELFE, and EPIPAGE-2 cohort studies

|  | **Unadjusted β-Coefficient [95%CI]** | **Adjusted β-Coefficient^a^ [95%CI]** |
| --- | --- | --- |
| *EDEN (n=1184)* |  |  |
| Obese (≥30kg/m²) | 0.33 [0.06, 0.60] | 0.32 [0.06, 0.59] |
| Overweight (25-29.9kg/m²) | 0.01 [-0.20, 0.22] | -0.05 [-0.26, 0.15] |
| Normal (18.5-24.9kg/m²) | REF | REF |
| Underweight (<18.5kg/m²) | 0.18 [-0.10, 0.46] | 0.16 [-0.12, 0.44] |
|  |  |  |
| *ELFE (n=10 889)* |  |  |
| Obese (≥30kg/m²) | 0.33 [0.24, 0.42] | 0.16 [0.07, 0.25] |
| Overweight (25-29.9kg/m²) | 0.14 [0.06, 0.21] | 0.05 [-0.02, 0.12] |
| Normal (18.5-24.9kg/m²) | REF | REF |
| Underweight (<18.5kg/m²) | 0.18 [0.07, 0.28] | 0.12 [0.02, 0.23] |
|  |  |  |
| *EPIPAGE-2 (n=2646)* |  |  |
| Obese (≥30kg/m²) | 0.33 [0.10, 0.55] | 0.19 [-0.04, 0.41] |
| Overweight (25-29.9kg/m²) | 0.07 [-0.12, 0.25] | 0.007 [-0.18, 0.19] |
| Normal (18.5-24.9kg/m²) | REF | REF |
| Underweight (<18.5kg/m²) | 0.30 [0.15, -0.01] | 0.24 [-0.06, 0.52] |

^a^Model adjusted for study centre (EDEN), maternal education, household income (ELFE only), household socioeconomic status (EPIPAGE-2), parity, sex, singleton pregnancy (EPIPAGE-2), psychological problems during pregnancy (EDEN+ELFE), maternal anxiety in pregnancy (EPIPAGE-2), maternal age at birth, maternal physical activity during pregnancy (EDEN+ELFE), maternal diet during pregnancy (EDEN+ELFE), maternal alcohol intake during pregnancy (EDEN+ELFE), maternal smoking during pregnancy, childcare at 2 years, gestational age, cause of prematurity (EPIPAGE-2), and child age at evaluation

**Supplementary Table 2.** Complete case analysis for peer relationship problems at 5.5 years in the EDEN, ELFE, and EPIPAGE-2 cohort studies

|  | **Univariate** | **Model 2** | **Model 3** | **Model 4** |
| --- | --- | --- | --- | --- |
| **Variable** | **Unadjusted OR [95%CI]** | **Adjusted OR^a^ [95%CI]** | **Adjusted OR^b^ [95%CI]** | **Adjusted OR^c^ [95%CI]** |
| *EDEN (n=1184)* | n=1165 | n=997 | n=969 | n=954 |
| Obese (≥30kg/m²) | 2.32 [1.42, 3.80] | 2.25 [1.35, 3.74] | 2.49 [1.42, 4.34] | 2.51 [1.41, 4.46] |
| Overweight (25-29.9kg/m²) | 1.21 [0.78, 1.88] | 1.14 [0.73, 1.78] | 1.29 [0.80, 2.01] | 1.25 [0.77, 2.02] |
| Normal (18.5-24.9kg/m²) | REF | REF | REF | REF |
| Underweight (<18.5kg/m²) | 1.25 [0.68, 2.29] | 1.18 [0.64, 2.19] | 1.23 [0.64, 2.38] | 1.33 [0.68, 2.60] |
|  |  |  |  |  |
| *ELFE (n=10 889)* | n=10 635 | n=10 013 | n=8957 | n=8528 |
| Obese (≥30kg/m²) | 1.81 [1.53, 2.14] | 1.47 [1.23, 1.77] | 1.43 [1.18, 1.74] | 1.34 [1.09, 1.65] |
| Overweight (25-29.9kg/m²) | 1.33 [1.16, 1.54] | 1.20 [1.03, 1.40] | 1.18 [1.00, 1.38] | 1.15 [0.97, 1.36] |
| Normal (18.5-24.9kg/m²) | REF | REF | REF | REF |
| Underweight (<18.5kg/m²) | 1.23 [1.00, 1.52] | 1.19 [0.95, 1.49] | 1.18 [0.93, 1.50] | 1.20 [0.94, 1.54] |
|  |  |  |  |  |
| *EPIPAGE-2 (n=2646)* | n=2458 | n=2298 | n=1594 | n=1394 |
| Obese (≥30kg/m²) | 1.70 [1.29, 2.24] | 1.53 [1.14, 2.06] | 1.54 [1.08, 2.20] | 1.50 [1.01, 2.25] |
| Overweight (25-29.9kg/m²) | 1.12 [0.87, 1.44] | 1.04 [0.79, 1.36] | 1.21 [0.87, 1.68] | 1.20 [0.83, 1.74] |
| Normal (18.5-24.9kg/m²) | REF | REF | REF | REF |
| Underweight (<18.5kg/m²) | 1.56 [1.08, 2.24] | 1.32 [0.90, 1.94] | 1.26 [0.78, 2.04] | 1.38 [0.82, 2.34] |

^a^Adjusted for study centre (EDEN), maternal education, household monthly income (ELFE), household socioeconomic status (EPIPAGE-2), parity, sex, psychological problems during pregnancy (EDEN+ELFE), maternal age at birth, singleton pregnancy (EPIPAGE-2)

^b^Additionally adjusted for maternal physical activity during pregnancy (EDEN+ELFE), maternal diet in pregnancy (EDEN+ELFE), maternal alcohol intake during pregnancy (EDEN+ELFE), maternal smoking during pregnancy, maternal anxiety during pregnancy (EPIPAGE-2)

^c^Additionally adjusted for gestational age, child age at evaluation, childcare at 2 years, cause of prematurity (EPIPAGE-2)

**Supplementary Table 3**. Cause of prematurity stratified analysis on the association between maternal pre-pregnancy body mass index and offspring peer-relationship problems at 5.5 years in the EPIPAGE-2 cohort (n=2646)

| **Variable** | **Unadjusted OR [95%CI]** | **Adjusted OR^a^ [95%CI]** |
| --- | --- | --- |
| *Preterm Labour (n=1214)* |  |  |
| Obese (≥30kg/m²) | 1.43 [0.84, 2.45] | 1.37 [0.78, 2.41] |
| Overweight (25-29.9kg/m²) | 1.05 [0.68, 1.61] | 0.94 [0.59, 1.50] |
| Normal (18.5-24.9kg/m²) | REF | REF |
| Underweight (<18.5kg/m²) | 1.26 [0.67, 2.39] | 1.06 [0.58, 1.94] |
|  |  |  |
| *PPROM^b^ (n=627)* |  |  |
| Obese (≥30kg/m²) | 0.69 [0.36, 1.32] | 0.50 [0.23, 1.06] |
| Overweight (25-29.9kg/m²) | 0.77 [0.41, 1.45] | 0.72 [0.36, 1.44] |
| Normal (18.5-24.9kg/m²) | REF | REF |
| Underweight (<18.5kg/m²) | 1.69 [0.76, 3.75] | 1.39 [0.59, 3.28] |
|  |  |  |
| *Vascular Pathology, Isolated Placental Abruption or Isolated IUGR^c^ (n=805)* |  |  |
| Obese (≥30kg/m²) | 2.82 [1.75, 4.57] | 2.57 [1.57, 4.21] |
| Overweight (25-29.9kg/m²) | 1.43 [0.89, 2.29] | 1.48 [0.91, 2.40] |
| Normal (18.5-24.9kg/m²) | REF | REF |
| Underweight (<18.5kg/m²) | 3.66 [1.49, 8.95] | 3.16 [1.22, 8.91] |

^a^Adjusted for maternal education, household socioeconomic status, parity, sex, maternal age at birth, singleton pregnancy, maternal smoking during pregnancy, maternal anxiety during pregnancy, gestational age, child age at evaluation, and childcare at 2 years

^b^PPROM: preterm premature rupture of the membranes

^c^IUGR: intrauterine growth restriction

**Supplementary Table 4**. Association between maternal pre-pregnancy body mass index and offspring peer-relationship problem trajectories from 3-8 years (n=1428), at 3 years (n=1307) and 8 years (n=875) in the EDEN cohort

| **Variable** | **Unadjusted OR [95%CI]** | **Model 1^a^ [95%CI]** | **Model 2^b^ [95%CI]** | **Model 3^c^ [95%CI]** |
| --- | --- | --- | --- | --- |
| *EDEN (3-8 Year Trajectory)* |  |  |  |  |
| Obese (≥30kg/m²) | 2.49 [1.34, 4.63] | 2.53 [1.33, 4.82] | 2.47 [1.29, 4.72] | 2.22 [1.15, 4.29] |
| Overweight (25-29.9kg/m²) | 1.59 [0.93, 2.73] | 1.49 [0.86, 2.57] | 1.55 [0.89, 2.70] | 1.51 [0.86, 2.65] |
| Normal (18.5-24.9kg/m²) | REF | REF | REF | REF |
| Underweight (<18.5kg/m²) | 1.23 [0.59, 2.54] | 1.11 [0.52, 2.35] | 1.04 [0.49, 2.24] | 1.10 [0.51, 2.36] |
|  |  |  |  |  |
| *EDEN (3 Years)* |  |  |  |  |
| Obese (≥30kg/m²) | 1.33 [0.81, 2.17] | 1.30 [0.79, 2.14] | 1.25 [0.75, 2.07] | 1.15 [0.69, 1.93] |
| Overweight (25-29.9kg/m²) | 0.97 [0.66, 1.43] | 0.94 [0.63, 1.40] | 0.91 [0.61, 1.36] | 0.88 [0.59, 1.33] |
| Normal (18.5-24.9kg/m²) | REF | REF | REF | REF |
| Underweight (<18.5kg/m²) | 0.84 [0.49,1.44] | 0.81 [0.47, 1.41] | 0.73 [0.41, 1.28] | 0.71 [0.40, 1.25] |
|  |  |  |  |  |
| *EDEN (8 Years)* |  |  |  |  |
| Obese (≥30kg/m²) | 1.48 [0.81, 2.71] | 1.61 [0.87, 2.98] | 1.58 [0.85, 2.95] | 1.58 [0.84 ,2.95] |
| Overweight (25-29.9kg/m²) | 1.16 [0.73, 1.86] | 1.18 [0.73, 1.92] | 1.17 [0.72, 1.91] | 1.15 [0.70, 1.88] |
| Normal (18.5-24.9kg/m²) | REF | REF | REF | REF |
| Underweight (<18.5kg/m²) | 1.52 [0.85, 2.69] | 1.38 [0.76, 2.49] | 1.29 [0.70, 2.37] | 1.33 [0.72, 2.44] |

^a^Adjusted for study centre, maternal education, parity, sex, psychological problems during pregnancy, maternal age at birth

^b^Additionally adjusted for maternal physical activity during pregnancy, maternal diet in pregnancy, maternal alcohol intake during pregnancy, maternal smoking during pregnancy

^c^Additionally adjusted for gestational age, child age at evaluation, childcare at 2 years

**
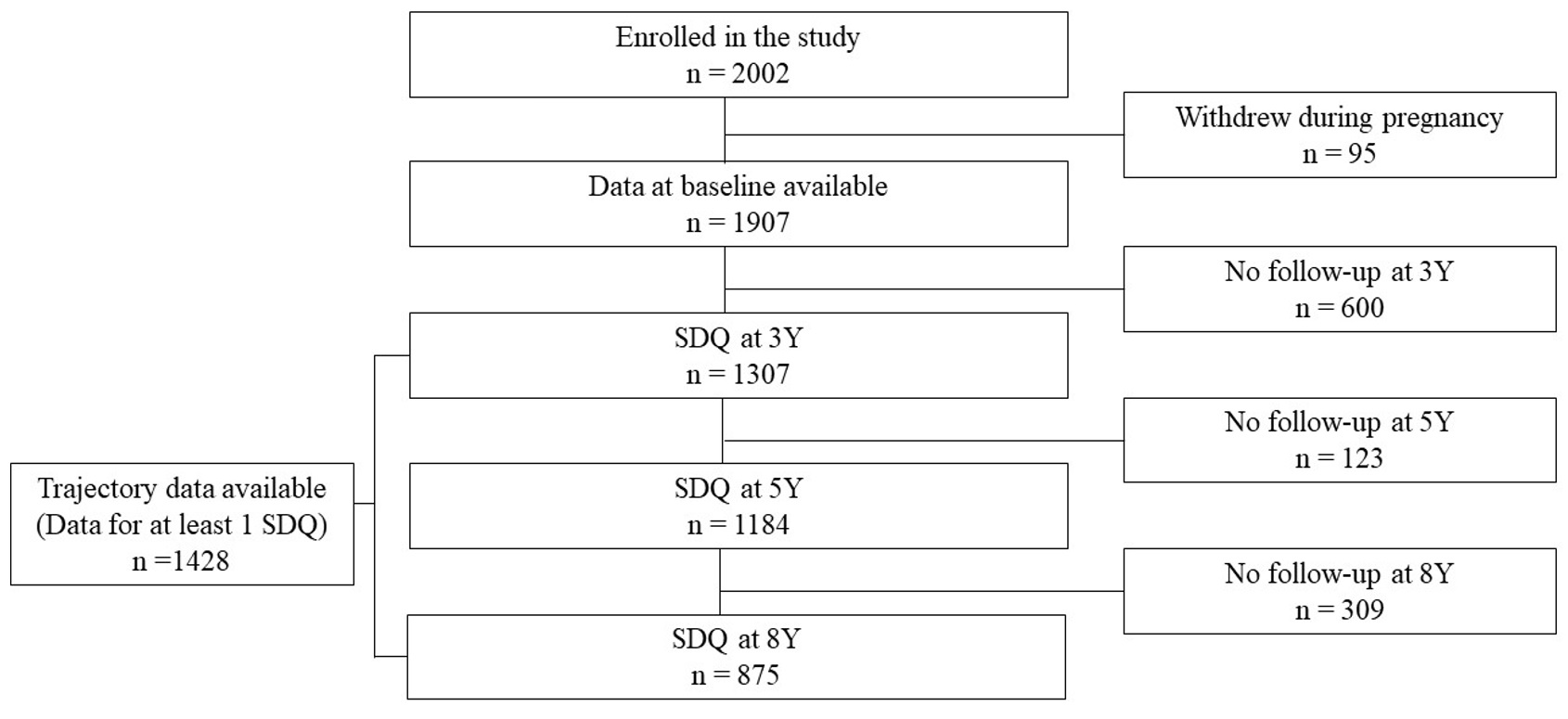
**

**Supplementary** **Figure 1a**. Flowchart of study participation in the EDEN cohort study


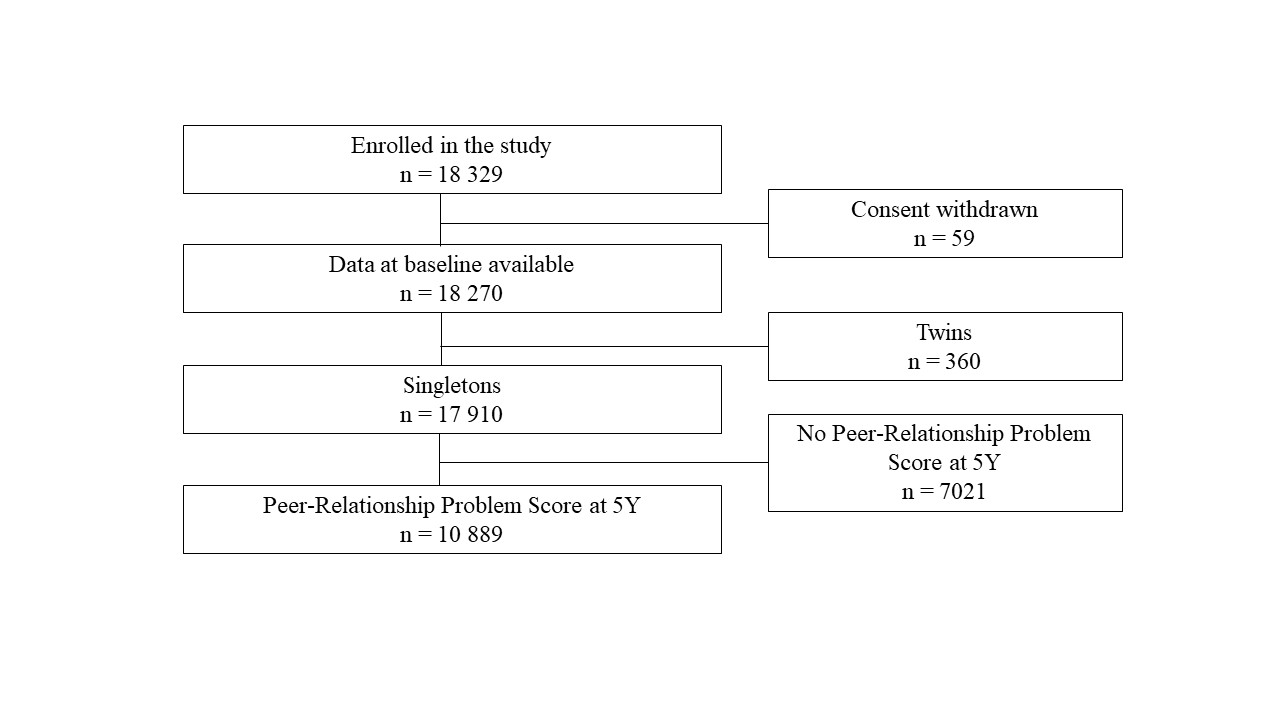


**Supplementary Figure 1b**. Flowchart of study participation in the ELFE cohort study


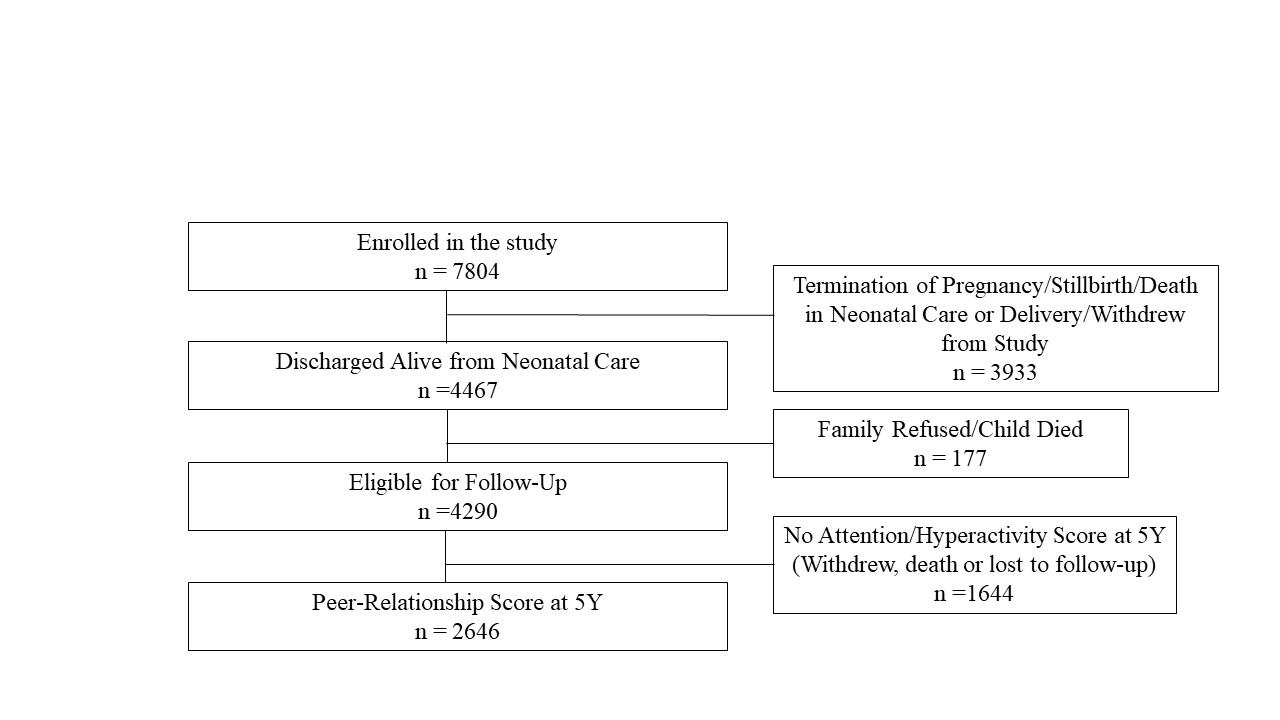


**Supplementary Figure 1c**. Flowchart of study participation in the EPIPAGE-2 cohort study
